# Assessment of Tie2-Rejuvenated Nucleus Pulposus Cell Transplants from Young and Old Patient Sources Demonstrates That Age Still Matters

**DOI:** 10.3390/ijms25158335

**Published:** 2024-07-30

**Authors:** Yuto Otani, Jordy Schol, Daisuke Sakai, Yoshihiko Nakamura, Kosuke Sako, Takayuki Warita, Shota Tamagawa, Luca Ambrosio, Daiki Munesada, Shota Ogasawara, Erika Matsushita, Asami Kawachi, Mitsuru Naiki, Masato Sato, Masahiko Watanabe

**Affiliations:** 1 Department of Orthopedic Surgery, Tokai University School of Medicine, 143 Shimokasuya, Isehara 259-1193, Japan; otani.20.dr@gmail.com (Y.O.); schol.jordy@gmail.com (J.S.); kahiko@is.icc.u-tokai.ac.jp (Y.N.); k.sako0626@gmail.com (K.S.); takayuki.warita@tunzpharma.co.jp (T.W.); s-tamagawa@juntendo.ac.jp (S.T.); l.ambrosio@unicampus.it (L.A.); kidai1149@yahoo.co.jp (D.M.); ogswr.a.817@gmail.com (S.O.); a.kawachi24062@gmail.com (A.K.); sato-m@is.icc.u-tokai.ac.jp (M.S.); masahiko@is.icc.u-tokai.ac.jp (M.W.); 2 Center for Musculoskeletal Innovative Research and Advancement (C-MiRA), Tokai University Graduate School, 143 Shimokasuya, Isehara 259-1193, Japan; 3 TUNZ Pharma Corporation, Osaka 541-0046, Japan; m-naiki@nippon-zoki.co.jp; 4 Department of Medicine for Orthopaedics and Motor Organ, Juntendo University Graduate School of Medicine, Tokyo 113-8421, Japan; 5 Operative Research Unit of Orthopaedic and Trauma Surgery, Fondazione Policlinico Universitario Campus Bio-Medico, 00128 Rome, Italy; 6 Research Unit of Orthopaedic and Trauma Surgery, Department of Medicine and Surgery, Università Campus Bio-Medico di Roma, 01128 Rome, Italy

**Keywords:** intervertebral disc, disc degeneration, cell therapy, regeneration, Tie2, progenitor cells, preclinical model, low back pain

## Abstract

Cell transplantation is being actively explored as a regenerative therapy for discogenic back pain. This study explored the regenerative potential of Tie2^+^ nucleus pulposus progenitor cells (NPPCs) from intervertebral disc (IVD) tissues derived from young (<25 years of age) and old (>60 years of age) patient donors. We employed an optimized culture method to maintain Tie2 expression in NP cells from both donor categories. Our study revealed similar Tie2 positivity rates regardless of donor types following cell culture. Nevertheless, clear differences were also found, such as the emergence of significantly higher (3.6-fold) GD2 positivity and reduced (2.7-fold) proliferation potential for older donors compared to young sources. Our results suggest that, despite obtaining a high fraction of Tie2^+^ NP cells, cells from older donors were already committed to a more mature phenotype. These disparities translated into functional differences, influencing colony formation, extracellular matrix production, and in vivo regenerative potential. This study underscores the importance of considering age-related factors in NPPC-based therapies for disc degeneration. Further investigation into the genetic and epigenetic alterations of Tie2^+^ NP cells from older donors is crucial for refining regenerative strategies. These findings shed light on Tie2^+^ NPPCs as a promising cell source for IVD regeneration while emphasizing the need for comprehensive understanding and scalability considerations in culture methods for broader clinical applicability.

## 1. Introduction

Low back pain (LBP) remains a pervasive global health concern, closely linked to intervertebral disc (IVD) degeneration [1,2,3]. The structural integrity of the IVD, particularly of the nucleus pulposus (NP), plays a pivotal role in maintaining spinal health [4,5,6,7,8]. Age-related disc degeneration is characterized by a discernible reduction in cellular abundance and their transition towards catabolic and senescent phenotypes with consequential alterations to the NP extracellular matrix (ECM) composition, posing a formidable challenge to therapeutic interventions [9,10,11,12,13,14,15,16]. Notably, at a relatively young age and early onset of degeneration, a rapid reduction in specific NP progenitor cells (NPPCs), marked by the angiopoietin-1 receptor tyrosine kinase (Tie2), has been observed and intricately linked to the progression of IVD degeneration [17,18]. This decline in NP cells has been identified as a promising target for designing impactful regenerative strategies [4]. Introducing de novo cells into the disc could potentially restore the balance of ECM production, thereby reinstating the biomechanical features of the IVD and thereby alleviating pain symptoms or alternatively alleviate the inflammatory state within the disc [19,20,21]. Several preclinical studies and ongoing clinical trials have suggested that reintroducing active cells into the NP can reduce LBP intensity and restore IVD features [20,21,22]. However, the research is still evolving to determine the optimal cell source [21,23] and processing methods that may support the large-scale production of high-quality and regenerative cell products [24,25,26,27].

Speculatively, the reintroduction of Tie2-expressing NPPCs hold great promise as a cell product for IVD repair and discogenic pain alleviation [28,29,30]. Unlike the most frequently applied mesenchymal stromal cells (MSCs), commonly sourced from, e.g., adipose tissue, bone marrow, and placenta [31,32,33,34], NPPCs are native to the IVD and intrinsically able to cope and thrive [23,35] within the harsh avascular disc tissue [36,37] and endure the significant load-bearing limits of the disc [38]. Moreover, Tie2^+^ NPPCs present remarkable stem cell-like features compared to their mature Tie2^−^ NP cell counterparts, including higher proliferation rates [17,39,40,41,42,43], increased differentiation potential [17,44,45,46,47], paracrine secretory activity [48,49,50], and enhanced ECM production [17,39,41,51]. Nevertheless, maintaining or expanding Tie2^+^ NPPCs in vitro poses a significant challenge [40]. NPPCs tend to rapidly lose their progenitor-like phenotype in culture, including Tie2 expression, rendering the manufacturing of market-scale NPPC-based transplantation products difficult [27,40,52]. Furthermore, the yields of Tie2^+^ NP cells from human disc explants are notably low, often compromised by age and the degeneration of the available NP source material [17,39]. Consequently, practical donor sources are generally limited to relatively young donors (<30 years old) undergoing surgery for lumbar disc herniation or traumatic vertebral fracture surgery, severely constraining the pool of tissue sources applicable for cell extraction [39,53].

As such, multiple studies have sought to optimize methods to isolate and culture Tie2+ NPPCs [41,44,47,53,54,55,56]. An optimized culture method previously described by Sako et al. [39] has demonstrated to significantly enhance the yield of Tie2^+^ NPPCs and their maintenance in culture. Nevertheless, this approach has only been tested on NP cells derived from young donors with a relatively early stage of disc degeneration, rather than on older patients experiencing more chronic and advanced degeneration. In this study, our aim was to assess the effectiveness of this culture method in increasing Tie2^+^ NPPC yields from older and more severely degenerated NP tissue explants. We sought to determine if the resulting cell products could maintain their regenerative potency, serving as potential alternative sources for creating NPPC-based transplantation products.

## 2. Results

### 2.1. NP Cell Surface Marker Expression

**Figure 1:**
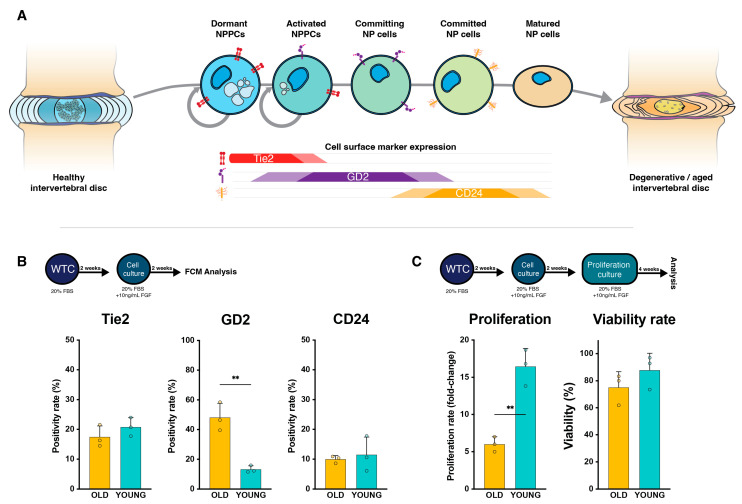
Assessment of nucleus pulposus progenitor cell (NPPC) surface markers. (**A**) Illustration depicting the presumed flow of NPPC activation and maturation [17] indicated by the expression of Tie2, GD2, and CD24, with receptor design based on previous work, [58], [59], and [60] respectively. (**B**) Flow cytometry outcomes and (**C**) assessment of cell proliferation indicated by fold-increase and cell viability. Bars represent mean values; dots represent single sample measurements; error bars indicate standard deviation. Statistical analysis performed through unpaired *t*-test. ** *p* < 0.01. Abbreviations: FBS: fetal bovine serum, FCM: flow cytometry analysis, FGF: fibroblast growth factor, NP: nucleus pulposus, NPPC: nucleus pulposus progenitor cell, WTC: whole tissue culture.

Surgical tissues were obtained upon Tokai University Institutional Ethics Review Board approval (17R173) and with the patients’ informed consent. Tissues were obtained from either young (<25 years old; YOUNG [*n* = 3]) or older (>60 years old; OLD [*n* = 3]) patients (Table 1), and subsequently cultured and processed as previously described [39]. This involved two weeks of whole tissue cultures (WTCs), subsequent cell isolation, and two additional weeks of monolayer culture. The resulting cells were analyzed through flow cytometry analysis [40] to determine their maturity and potency state [17] (Figure 1A). Surprisingly, both young and old donors led to similar Tie2 positivity rates (YOUNG: 20.8 ± 3.2%, OLD 17.5 ± 3.7%; *p* = 0.300). Contrarily, disialoganglioside (GD2) positivity was higher in OLD samples (48.2 ± 9.6%) compared to YOUNG (13.3 ± 2.4%, *p* = 0.004) NP cell populations. While CD24 expression was slightly increased in YOUNG samples, between-group differences were not statistically significant (11.5 ± 5.9% vs. 10.0 ± 1.2; *p* = 0.682) (Figure 1).

**Table 1:** Tabular overview of the demographic characteristics of the IVD donor samples along with Tie2, GD2, and CD24 expression rates.

Category	No	Age (Years)	Sex	Pfirrmann Grades [57]	Level(s)	Indication	Tie2 (%)	GD2 (%)	CD24 (%)
YOUNG	1	16	M	2	L4–L5	LDH	20.7	12.3	17.8
	2 ^†^	14	F	/	/	LDH	24.0	16.0	6.1
	3	23	M	3	L5-S1	LDH	17.7	11.6	10.6
Average		18 ± 5					20.8 ± 3.2	13.3 ± 2.4	11.5 ± 5.9
OLD	4	64	M	4	L3–L5	LCS	14.5	46.4	10.4
	5	65	M	5	L3–L5	LCS	21.6	58.5	10.9
	6	66	F	4	L4–L5	LCS	16.3	39.6	8.6
Average		65 ± 1					17.5 ± 3.7	48.2 ± 9.6	10.0 ± 1.2
*p*-value *		<0.0001					0.300	0.004	0.682

* Unpaired *t*-test ^†^ Sample obtained from affiliated institute; medical records not fully accessible. Abbreviations: LCS: Lumbar canal stenosis, LDH: Lumbar disc herniation.

### 2.2. NP Cells’ Proliferation Rate and Viability

The harvested cells from our Tie2-enhancing culture conditions were cultured for an additional 4 weeks to determine their long-term proliferative potential. Despite presenting similar Tie2 positivity rates, the YOUNG NP cells exhibited a significantly higher proliferative capacity (16.4 ± 2.4-fold increase) compared to their OLD NP cell counterpart (6.0 ± 1.0-fold increase, *p* = 0.002), with similar viability rates (*p* = 0.266) (Figure 1C).

### 2.3. Regenerative Potential Assessment

**Figure 2:**
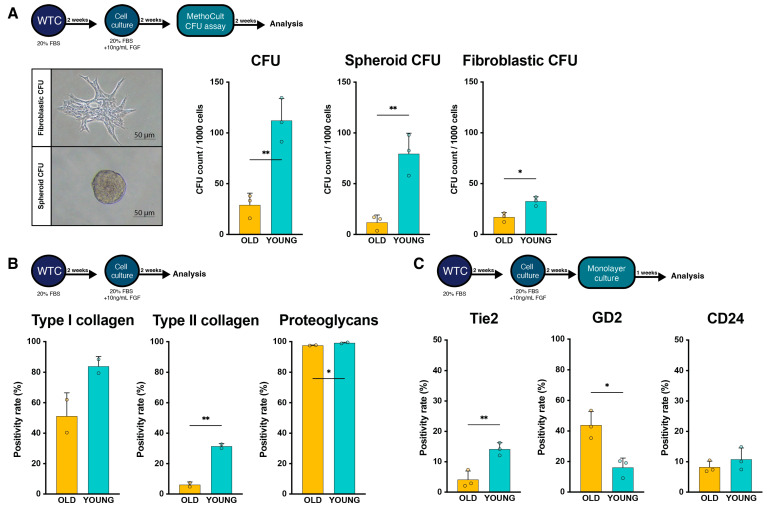
Functionality assessment of obtained nucleus pulposus (NP) cells [40]. (**A**) Pictures of typical fibroblastic colony-forming units (CFUs) and spheroid CFUs as presented in previous work [40]. Scale bar represent 50 µm. Graphs show the rate of the total CFUs, fibroblastic CFUs, and spheroid CFUs (Left to right). (**B**) The rate of positive cells for type I collagen, type II collagen, and proteoglycans as assessed through flow cytometry. (**C**) Re-assessment of Tie2, GD2, and CD24 positivity of obtained cells cultured for an additional week. Bars represent mean values; dots represent single sample measurements; error bars indicate standard deviation. Statistical analysis performed through unpaired *t*-test. * *p* < 0.05, ** *p* < 0.01. Abbreviations: FBS: fetal bovine serum, FGF: fibroblast growth factor, NP: nucleus pulposus, WTC: whole tissue culture.

Functional characterization, assessed through the colony formation assay (CFA) in methylcellulose media, revealed an increased rate of colony-forming units (CFUs) in the YOUNG samples compared to the OLD samples (Figure 2A). Despite displaying similar Tie2 positivity rates, the YOUNG samples exhibited a higher yield of CFUs compared to OLD samples (*p* = 0.004). More specifically, the rate of spheroid CFUs was notably elevated in the YOUNG group, reaching 79.6 *±* 22.5 per 1000 cells, as opposed to 12.0 *±* 6.3 per 1000 cells in the OLD group (*p* = 0.006). Also, the resulting fibroblastic CFUs per 1000 cells from YOUNG cells significantly surpassed the yields from OLD samples (*p* = 0.012). This suggests that the age of the donors significantly influenced the functional progenitor cell-like phenotype, despite comparable Tie2 expression rates.

The cellular yields were further characterized for their ECM production potential using flow cytometry analysis (Figure 2B). The YOUNG samples demonstrated a higher proportion of cells producing type I collagen (*p* = 0.108) and type II collagen (*p* = 0.005) compared to the OLD samples. Notably, regardless of age, nearly all isolated cells exhibited positive staining for proteoglycans, with a significantly higher rate reported in the YOUNG cells (*p* = 0.044).

### 2.4. Change in Cell Surface Markers with Extended Culture

A separate fraction of the obtained cells that was prepared for analysis and transplantation was cultured for an additional week to assess the retention of NPPC phenotypes. YOUNG cells showed a slight change in Tie2 positivity from 20.8 ± 3.2% to 14.2 ± 2.1%, compared to a significant decline from 17.5 ± 3.7% to 4.1 ± 2.9% in OLD samples (*p* = 0.008) (Figure 2C). GD2 expression did not markedly change following extended culture within each group, and positivity rates remained significantly higher in the old samples (*p* = 0.012). Likewise, CD24 positivity rates were kept constant and showed no difference between the YOUNG and OLD samples.

### 2.5. Correlation Assessment

**Figure 3:**
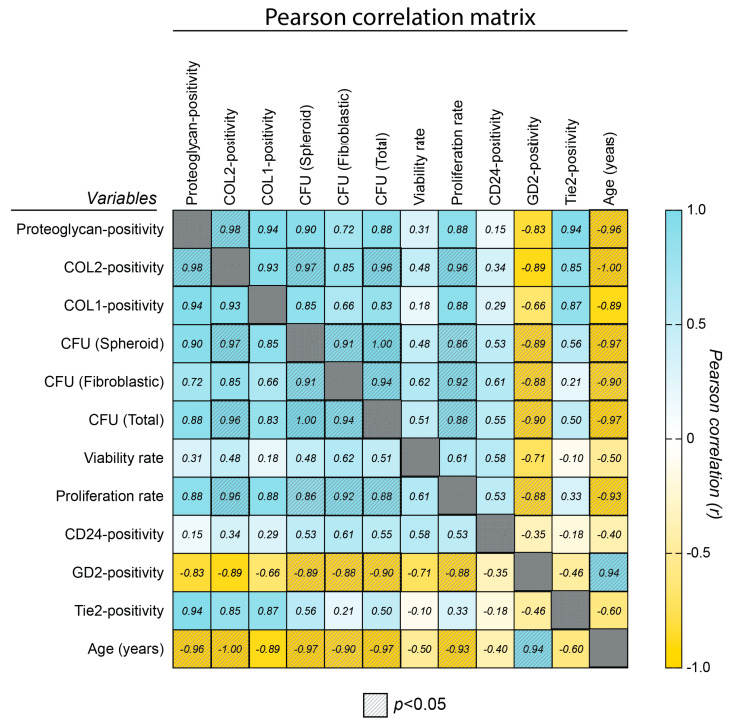
Pearson correlation matrix of the variables assessed in vitro. Diagonally striped boxes indicate significant (*p* < 0.05) correlations. Abbreviations: CFU: colony-forming units, COL-1: type I collagen, COL-2: type II collagen.

A Pearson correlation assessment (Figure 3) was employed to assess the link between age, surface markers, progenitor cell features, and ECM production potential and determine a link between markers and outcomes. These underscored a significant and negative correlation of NPPC features (including CFU rates, proliferation rates, and ECM potential) with both donor age and GD2 positivity. Moreover, although Tie2 positivity rates were similar between the YOUNG and OLD groups following our optimized culture, the slight trend of enhanced Tie2 in YOUNG samples still allowed for the identification of strong correlation trends between Tie2 positivity and proteoglycan positivity (R = 0.95, *p* = 0.060), type II collagen positivity (R = 0.85, *p* = 0.152), and type I collagen production (R = 0.87, *p* = 0.110). This emphasized Tie2 as a potent regenerative cell surface marker within NP cells, aligning with previous observations [39].

### 2.6. Transplantation Surgery

**Figure 4:**
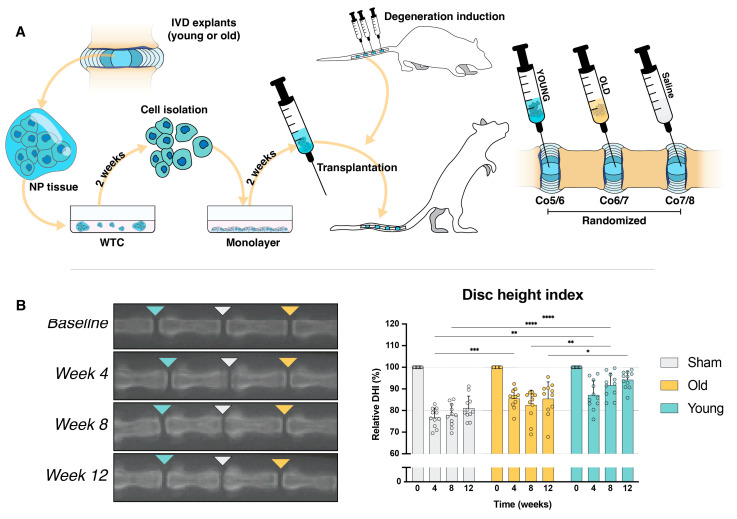
Examination of cell transplantation products derived from old and young tissue sources. (**A**) Illustration depicting the general procedure of cell isolation, culture, and subsequent transplantation. (**B**) Assessment of the radiographic images and disc height index (DHI) measurements at 4, 8, and 12 weeks post-transplantation. Arrow-head indicates a disc treated with either Young donor cells (Teal), Old donor cells (Orange), or Sham saline solution (Light-grey); bars represent mean values; dots represent single sample measurements; error bars indicate standard deviation. * *p* < 0.05, ** *p* < 0.01, *** *p* < 0.005, and **** *p* < 0.001. Abbreviations: NP: nucleus pulposus, WTC: whole tissue culture. The surgical procedure and transplantation were successfully performed without any complications; however, one rat was excluded due to a technical error occurring following surgery. Body weight and general behavioral assessments revealed no complications as part of the surgical intervention.

Cell transplantation products generated from both YOUNG and OLD donor sources (Table 1) were applied as an off-the-shelf transplantation product [61], applied in a well-established rat coccygeal-induced disc degeneration model [48,62] (Figure 4A). Twelve male rats (13 weeks of age) underwent disc degeneration induced by needle puncture at coccygeal level (Co)5–Co6, Co6–Co7, and Co7–Co8. These rats were then directly treated with either 25 µL of phosphate-buffered saline (PBS) solution (Sham) or 25 µL of 4 × 10^6^ NP cells mL^−1^ from either YOUNG or OLD donor sources (Table 1). The locations of the three different transplantation products were randomized over the three disc levels. Co4/5 and Co8/9 were used throughout as a healthy reference control (Figure 4A).

Just prior to and at 4, 8, and 12 weeks following degeneration induction and transplantation surgery, radiographic images of the coccygeal region were captured and analyzed for changes in the relative disc height index (DHI) compared to the baseline of each individual rat, i.e., their respective disc height measured before the induction of degeneration (Figure 4B). The measurements revealed a significant (*p* < 0.001) decline in the DHI for the Sham-treated discs at 4 weeks, which decreased to 76.7 *±* 4.4%, with a slight recovery to 81.1 *±* 5.5% at 12 weeks post-operation. Discs treated with OLD NP cells significantly (*p* < 0.001) mitigated DHI loss compared to the Sham control at 4 weeks but did not show improvement at later time points, resulting in a relative DHI of 85.5 *±* 7.8% at week 12. In contrast, the YOUNG cell-treated discs significantly (*p* = 0.001) limited the induced deterioration at week 4 compared to the Sham-treated discs and continuously improved the DHI, resulting in a significantly enhanced DHI at week 12 of 94.2 *±* 3.9%, outperforming both the Sham- (*p* < 0.001) and OLD NP cell (*p* = 0.012)-treated discs.

**Figure 5:**
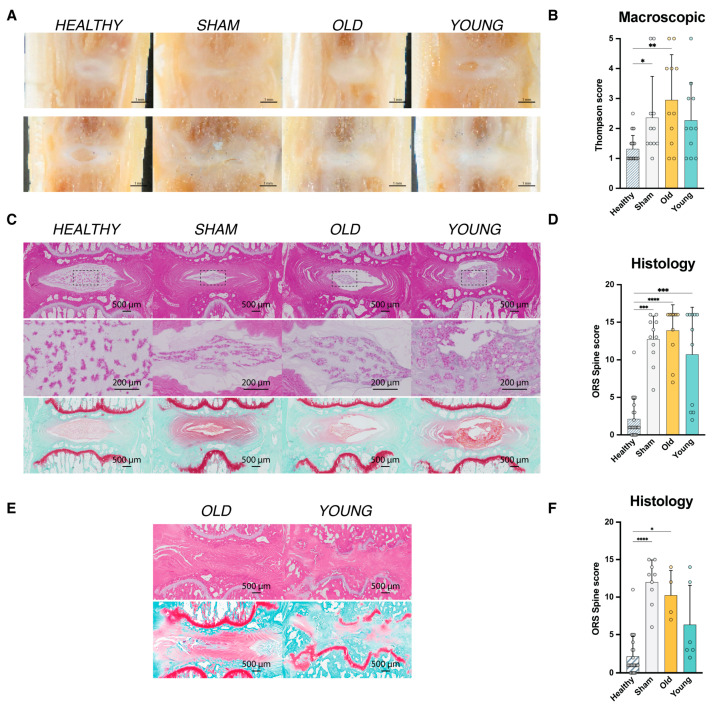
Macroscopic and histological assessment of experimental intervertebral discs (IVDs) 12 weeks post-transplantation procedure. (**A**) Impression of macroscopic results of explanted IVDs. Scale bar represents 1 mm. (**B**) Graphical representation of assigned Thompson scores [63] to explanted IVDs. Bars represent mean values; dots represent single sample measurements; error bars indicate standard deviation. * *p* < 0.05, ** *p* < 0.01. (**C**) General impression of histological outcomes as stained by hematoxylin/eosin (top row) and safranin-O/Fast green (bottom row). (**D**) Gradings of explanted discs through the rat-specific ORS spine histopathological scheme [64]. Bars represent mean values; dots represent single sample measurements; error bars indicate standard deviation. *** *p* < 0.005 and **** *p* < 0.001. (**E**) Examples of discs presenting fully disorganized IVD tissue. (**F**) Gradings of explanted discs, excluding treated disc presenting with full collapse through the rat-specific ORS spine histopathological scheme [64]. Scale bar represents 500 µm in macroscopic images and 200 µm in magnified panels. Bars represent mean values; dots represent single sample measurements; error bars indicate standard deviation. * *p* < 0.05 and **** *p* < 0.001.

At week 12 post-transplantation, the rats were sacrificed, and both experimental and control discs were prepared for further macroscopic and histological analysis. Thompson scoring [63] of macroscopic images revealed deterioration in disc morphology for all experimental discs compared to their healthy control discs, though only Sham and OLD conditions presented a significant worsening in average classification grades (Figure 5A,B).

Next, hematoxylin/eosin- and safranin-O/fast-green-stained sections were graded according to recommendations of the Orthopedic Research Society (ORS) [64]. The results match the macroscopic observations, with all conditions showing overall worsening in histological organization, with the YOUNG condition exhibiting a noticeable trend of improvement compared to the Sham and OLD conditions. However, a relatively large fraction of samples exhibited complete disorganization (scores > 15 points) across all treatment conditions, despite no apparent loss in DHI (Figure 5E). We considered these deteriorated discs potential technical errors, and when excluded from the analysis, the trend of outcomes suggested enhanced outcomes for YOUNG samples compared to OLD conditions, and the OLD condition consequently showing enhanced outcomes compared to the Sham control (Figure 5E,F).

**Figure 6:**
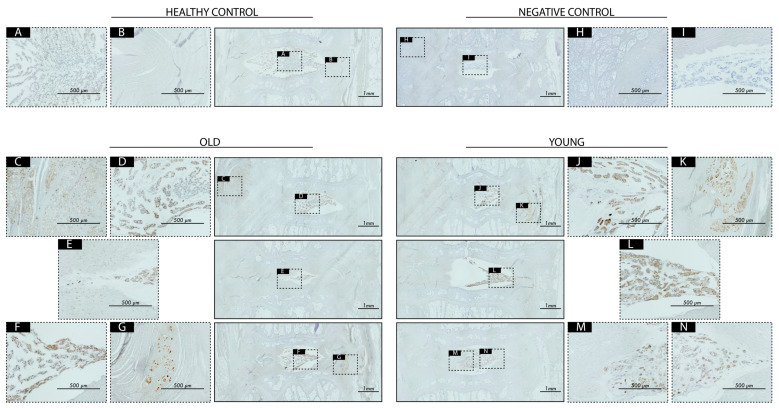
Immunohistochemistry-mediated detection of HLA for human NP cell tracking (**A**–**N**) indicates specific magnified regions of interest. Note brown (HLA-indicative) staining both within disc (**D**–**F**,**J**,**L**–**N**) and outside the disc (**C**,**G**,**K**). Scale bars represent 1000 µm in macroscopic view or 500 µm in magnified panels as indicated. Negative control involves stained section lacking primary antibodies.

For the disc explants that scored low (i.e., with better preserved disc organization) in the histological scoring, immunohistochemistry (IHC) staining against human leukocyte antigen (HLA) was performed to detect remnant human NP cells within the rat IVDs, which has been shown to be an effective method to trace xenogenically transplanted human NP cells in a previous work [61]. Firstly, we noted no evident immuno-positivity in NP, AF, or other regions of our non-treated discs. For both discs treated with OLD and YOUNG donors, we found HLA positivity present, predominantly located in the NP and the inner AF regions (Figure 6). Notably, in some slides, the transplanted human cells were detected beyond the IVD perimeters, suggesting that a portion of the transplanted cell volume had leaked out of the disc (Figure 6).

## 3. Discussion

Tie2 is a transmembrane receptor primarily expressed on endothelial cells involved in angiogenesis and vascular stability by interacting with its ligands, angiopoietin-1 and -2 [65,66]. The activation of Tie2 signaling regulates endothelial cell survival, vascular maturation, and blood vessel maintenance, contributing to the dynamic balance of tissue homeostasis and angiogenic processes [67,68,69]. This receptor’s unique attribute of promoting a stem cell-like phenotype in endothelial cells underscores its significance in orchestrating regenerative processes within the vasculature [69,70,71]. The discovery of Tie2^+^ cells within the otherwise avascular IVD [36] has delineated a specific NP cell population, presenting a high proliferation and a progenitor cell-like phenotype [17], and has warranted extensive research for its potential application in regenerative medicine [21]. Multiple methodologies have been explored to increase yields or augment Tie2 expression in NPPCs cultured in vitro. Methods have focused on culture conditions [40,41,44,47,53,55], sorting methods [40], and cryopreservation techniques [53,54,72], all indicating that processing adjustments can significantly enhance Tie2^+^ NPPC retention. The optimization of culture conditions, as established by Sako et al. [39], has shown promise in retaining Tie2^+^ NPPCs in vitro and is currently being applied for the commercial development of NPPCs as cellular therapeutics. Nevertheless, these methods are commonly applied only on NP cells derived from relatively young donor sources.

The culture methods described by Sako et al. [39], including WTC and subsequent fibroblast growth factor (FGF)-2-supplemented monolayer culture, were able to yield high rates of Tie2^+^ NP cells from young donors (on average 20.8% positivity), as expected. Surprisingly, these methods were able to yield similar Tie2 positivity rates for NP cells derived from senior patients, emphasizing the potential of culture methods to promote the retention of Tie2 positivity. Moreover, considering the average rate of Tie2^+^ cell yields from individuals above the age of 40 years is expected to be around 2% [17,40], the impact of this approach seems to extend beyond just retaining Tie2^+^ cells and instead promotes the rejuvenation of the cell population. Nevertheless, despite the strong Tie2 positivity increase and similar proportions of CD24-expressing cells, NP cells derived from older sources differed from their younger controls, as observed in their different GD2 positivity rates. GD2 has been implicated in processes associated with cell proliferation, adhesion, and migration [73,74], while CD24 is more generally involved in cell adhesion, migration, and differentiation [75,76]. The distinctive role of GD2 and CD24 with NP cells remain obscure [77]; however, GD2 expression has been linked with the cellular commitment of Tie2^+^/GD2^−^/CD24^−^ NPPC towards the committed CD24^+^ NP cells [17], eventually expressing the fully matured CD24^−^ NP cells (Figure 1A). Therefore, despite older NP cells leading to Tie2^+^ NP cells, there seems to be a higher fraction of Tie2^+^ NP cells already committed to a more mature phenotype than NPPCs derived from younger donors.

These considerations could help to explain the evident functional differences in OLD versus YOUNG samples, including reduced colony formation, ECM production potential, as well as limited regenerative potential in our rat disc degeneration model. Despite being able to augment the proportion of Tie2 positivity in the older samples, the lack of consequential regenerative potential is not unexpected. Aging and a chronic catabolic environment (e.g., as part of disc degeneration [16,78,79,80]) are known factors that may promote epigenetic alterations and DNA damage [81,82,83], which in turn can temper the potential of stem and progenitor cells [84,85,86]. These (epi)genetic changes in stem/progenitor cells are linked with various age-related pathophysiology and could be themselves targets for new therapeutic strategies [87,88,89]. Whether these DNA modifications are responsible for the different functionalities of the old versus young NPPCs remains a target for future research.

### Limitations and Considerations

Our cellular transplant was able to maintain DHI levels, yet the histological observation did not report clear beneficial results for the cell transplants sourced from neither donor sources. While YOUNG cells displayed a higher in vitro potential compared to the OLD condition, this superiority did not manifest in an evident enhancement in tissue reorganization. This incongruity underscores the urgency in addressing fundamental questions within the ongoing discourse on cell therapy for disc degeneration [21,31,90]. A pivotal query centers around the fate of transplanted cells within the disc microenvironment, i.e., whether transplanted cells actively integrate into the disc, contributing directly to ECM production and fostering disc homeostasis in a sustained manner, or if their impact is predominantly transient and mediated through paracrine signaling [91,92]. The prior successful application of MSCs into neighboring vertebrae [49,93], systematic MSC infusion [93,94], and the efficacy of extracellular vesicles-based products [48,95,96,97,98] underscore the potential role of paracrine signaling in mediating therapeutic effects. Moreover, concerns persist regarding the additional cellular demand introduced by the transplanted cells [31,99,100], potentially exacerbating the strain on the already hypoxic and harsh disc environment [101,102,103]. These complexities are further compounded by the acute nature of our disc degeneration model [62,104,105,106,107], which may not fully reflect the gradual and chronic degeneration progression observed in human cases [108,109]. Moreover, the rat IVD is composed of different cellular populations [14,28,62,99] and represents different disc size and composition [49,62,64,110,111], which also require careful consideration. It is important to note that our animal model exclusively involved male specimens. Given the known differences in disc degeneration and associated pain between sexes [112,113,114,115], future research should investigate whether female rats exhibit similar responses [116].

Finally, through our HLA-IHC staining, we observed that a considerable volume of transplanted cells leaked out of the disc. In our study, we employed a total transplant volume of 25 µL, which may be too large a volume for transplantation into a coccygeal rat IVD [62,99]. The comprehensive review by Barcellona et al. [62] suggested that “volumes of 2–5 µL may be best suited for intradiscal delivery” for rats. This will be an optimization future studies will consider. Moreover, with the consideration of clinical translation, the leakage of cells is considered suboptimal, as it may promote undesired tissue differentiation elsewhere, e.g., osteophyte formation [31,117], or promote a graft-versus-host response [118]. However, despite this suboptimal aspect, our study demonstrated evident beneficial effects on DHI maintenance for the cell-treated discs. Importantly, at the current follow-up stage, no evident osteophyte formation attributed to our NP cell products was detected. Additional work would also likely benefit from exploring strategies tackling damage of the anulus fibrosus and cartilaginous endplate [8,119,120,121]. While clinical cell therapy studies have demonstrated positive outcomes, the need for large-scale, high-quality randomized controlled trials remains imperative for a conclusive understanding of the therapeutic efficacy of cell transplantation for disc degeneration [19,122,123].

In conclusion, this study sheds light on the potential of Tie2^+^ NPPCs as a promising cell source for IVD regeneration, although our work suggests that older tissue sources are likely suboptimal for NPPC production. The findings underscore the need for further investigations to unlock the full therapeutic potential, addressing scalability challenges and exploring alternative methods. As the field of regenerative medicine progresses, understanding the intricacies of Tie2^+^ NPPCs and their role in disc homeostasis remains a dynamic area for continued research.

## 4. Materials and Methods

### 4.1. Human NP Cell Isolation and Culture

Approval for this study was granted by the Institutional Review Board for Clinical Research at Tokai University (application number: 17R-173), ensuring that all research procedures described in the study complied with the ethical and safety standards set by our institution. The study included the collection of NP tissue samples from 6 patients (Table 1). Disc tissues were categorized as YOUNG if the patient age was below 25 years or OLD if above the age of 60 years. Prior to tissue collection, all patients provided informed written consent, signifying their agreement to the utilization of surgical waste for research purposes. In the case of patients under the age of 18 years, informed consent was obtained from their parent(s) or legal guardian(s).

According to recommendations set forth in previous work [39,40], the collected disc tissue was washed with saline to remove all traces of blood. Next, through macroscopic examination, NP tissue was carefully selected and subsequently cut into 3–5 mm diameter pieces using scissors and scalpels. The NP fragments were directly placed onto a specialized NPPC-optimized medium, involving a blend of MEMα (32%), DMEM (48%), and fetal bovine serum (FBS; 20%), commercially developed by TUNZ Pharma Co., Ltd. (Osaka, Japan) [39]. The tissue was directly seeded in polystyrene 6-well plates (IWAKI, Tokyo, Japan), with approximately 0.3 g of NP tissue in 3 mL of culture media per each well (equivalent to about 32 mg/cm^2^). The tissue fragments underwent a 14-day culture at 37 °C in a physioxia (5% CO_2_ and 5% O_2_) environment [124,125], without media replenishment (Figure 1A).

Following two weeks of WTC, the NP fragments were carefully transferred to a 50 mL conical tube and centrifuged at 1200 rpm for 5 min at 4 °C. Following centrifugation, the supernatant was discarded, and the tissue was resuspended in 20 mL of a solution containing 1:1 TrypLE Express (Thermo Fisher Scientific, Tokyo, Japan) and 10% (*v*/*v*) FBS, minimal essential medium α (MEMα, Fujifilm Wako Pure Chemical Corporation, Osaka, Japan). The suspension underwent digestion under gentle shaking at 37 °C for 1 h. After confirming tissue dissolution, the sample was centrifuged at 1800 rpm for 5 min. The collected tissue underwent additional digestion in a blend of 15 mL, comprising 10% (*v*/*v*) FBS, αMEM, and 5 mL of collagenase P (0.25 mg mL^−1^, Roche, Basel, Switzerland), and was then incubated for 2 h at 37 °C. After digestion, the suspension underwent centrifugation once more, followed by resuspension in 20 mL of 10% (*v*/*v*) FBS MEMα. Finally, the suspension was filtered through a 40 μm cell strainer (Corning, Corning, NY, USA). Subsequently, the derived cells were cultured on poly-L-Lysine-coated plasticware (IWAKI) for an additional two weeks in identical media supplemented with 10 ng mL^−1^ FGF-2 (PeproTech, Cranbury, NJ, USA) in a 5% CO_2_ and 5% O_2_ environment. NP cells were seeded at a density of 30,000 cells per plate (545.5 cells cm^−2^). Following the indicated culture periods, the cells were harvested by rinsing with PBS once and treating with TrypLE Express at 37 °C for 5 min. The collected cells were then suspended in 10% (*v*/*v*) FBS MEMα, counted, and immediately utilized for experimentation or subjected to cryostorage (see below).

### 4.2. Flow Cytometry Analysis

NP cells were examined using a FACS Calibur flow cytometer (BD Biosciences, Franklin Lakes, NJ, USA), in accordance with previously established protocols [39,40,41]. In the evaluation, a propidium iodide-negative gate was applied to exclusively analyze living cells. Flow cytometry was utilized to assess the fractions of NPPC markers, including Tie2 and GD2 [40], NP cell marker CD24 [40,126], as well as intracellular ECM precursors for type I collagen, type II collagen, and proteoglycans [127,128].

Staining for cell surface markers were performed through mouse anti-human Tie2 antibody with conjugated APC (monoclonal IgG1, FAB3131A [R&D Systems, Minneapolis, MN, USA]), mouse anti-human GD2 antibody with conjugated PE (monoclonal IgG2a, 562100 [BD Biosciences]), and mouse anti-human CD24 antibody conjugated with FITC (monoclonal IgG2a, 555427 [BD Biosciences]). Intracellular staining was performed by fixing cells in formalin and using the IntraPrep Permeabilization Reagent (Beckman Coulter, A07803). Subsequent staining was performed using mouse anti-human type I collagen (monoclonal IgG2a, F-56 [Kyowa Pharma Chemicals Co., Ltd., Takaoka, Japan]), mouse anti-human type II collagen (monoclonal IgG1, F-57 [Kyowa Pharma Chemicals Co., Ltd.]), and mouse anti-human cartilage-proteoglycan antibody (monoclonal IgG1, EFG-4 MAB2015 [Sigma-Aldrich, Burlington, MA, USA]). Secondary staining was performed with goat anti-mouse antibody conjugated with FITC (polyclonal IgG1, 349031 [BD Biosciences]). The staining and flow cytometry protocols for intracellular proteoglycans and type I and type II collagen were performed as previously described [41].

### 4.3. Proliferation Assessment and Viability Assay

Following previously described methods, the isolated NP cells from YOUNG and OLD donors were cultured for an additional 4 weeks under identical conditions and thereafter harvested using TrypLE express. The total cell number and viability rates were thus assessed using Trypan blue (Fujifilm Wako Pure Chemicals Corporation, Osaka, Japan, 0.4% *w*/*v*) exclusion methods in a hemocytometer. To determine NPPC surface marker expression changes with an extended culture period, the collected cells were seeded and cultured for an additional week, and Tie2, GD2, and CD24 expression was re-evaluated as described above [40].

### 4.4. Methylcellulose-Based Colony-Forming Unit Assay

In accordance with established methods [17], following optimized culture methods [39], a total of 1000 YOUNG or OLD NP cells were encapsulated in 1 mL Methocult (A methylcellulose-based hydrogel; ST-04230 STEMCELL Technologies, Vancouver, BC, Canada) and cultured for a duration of 10 days at 37 °C with 5% O_2_ and 5% CO_2_ as a CFA. Subsequently, the total number and the number of fibroblastic and spheroid CFUs per 1 mL of MethoCult were manually counted using an inverted phase-contrast microscope (Model ECLIPSE Ti2-U; Nikon Corporation, Tokyo, Japan).

### 4.5. Rat Disc Degeneration Model and Cell Transplantation

Yielded NP cells were frozen in CryoStor^®^-10 (CS10; STEMCELL Technologies), according to Sako et al. [53], and stored in liquid nitrogen until transplantation and used as off-the-shelf transplantation products. At time of transplantation, the cells were taken from their cryopreserved state and kept on ice to thaw just prior to injection.

The animal experiments were reviewed and approved by the Tokai University Institutional Ethics Review Board under the ID #215005. Twelve male Sprague Dawley rats (MIZUSETSU, Sapporo, Japan) were kept under controlled environment. Following acclimatation and the acquisition of baseline data, disc degeneration was induced by annular puncture of 3 coccygeal discs as previously described [49]. Briefly, animals were placed on a heated pad to prevent hypothermia, sedated with continuous 2.5% isoflurane inhalation, and placed in supine position. The surgical area was carefully sterilized with 70% ethanol and disc degeneration was induced from Co5-Co6 to Co7-Co8 by percutaneous annular puncture and subsequent aspiration using a 22G, 1”, 0.7 × 25 mm needle (TERUMO, Tokyo, Japan) combined with a 5 mL syringe (ss-05Sz TERUMO), as described in previous studies [49]. Central placement was carefully controlled through fluoroscopic navigation.

Immediately after disc puncture, the thawed cell suspension was taken and directly mixed 1:1 with ARTZ Dispo^®^ (Seikagaku Corporation, Tokyo, Japan), which is composed of 1% sodium hyaluronate solution (HA; Average MW: 5.0 × 10^5^ to 1.2 × 10^6^ Da, concentration: 25 mg 2.5 mL^−1^) to create a final product containing 4 × 10^6^ cells mL^−1^ in CS10/0.5%HA and directly used as a transplantation product. ARTZ Dispo^®^ formulation has proven effective as an intra-articular agent for knee osteoarthritis [129,130] and has been validated for its in vitro effectiveness on attenuating DMSO-induced cytotoxicity on NP cells in a previous work [54]. The central needle placement using a fluoroscopic intensifier was confirmed and either 25 μL of PBS (Sham) or 25 µL of HA/CS10-suspended 1 × 10^5^ NP cells from YOUNG or OLD donors were administered through a 27G gauge insulin syringe (TERUMO). Each rat received one of each treatment types in one of the three degenerated levels. The allocation of treatment types was randomly assigned using the Excel (version 16.78.3, Microsoft Corp., Redmond, WA, USA) randomization function. Each of the three donor source materials (Table 1) were separately applied into 4 rats each. Co4-Co5 and Co8-Co9 levels were kept as healthy reference controls throughout the study. Subsequent to the injection, the rats received buprenorphine hydrochloride (0.05 mg kg^−1^; Otsuka Pharmaceutical, Tokyo Japan) in 0.2 mL for pain alleviation. Rats were treated with 100 µL of immunosuppressant tacrolimus hydrate (Astellas Pharma Inc., Tokyo, Japan) at 200 mg/kg/day intramuscularly for up to 14 consecutive days starting two days before surgery. Following removal from sedation, the rats recovered under a heat lamp, and after confirmed convalescence, the rats were placed back into their housing area. The body weight of rats was monitored continuously.

### 4.6. Radiographic Assessment

Radiographic evaluation was performed at baseline and 4, 8, and 12 weeks post-operatively. Coccygeal region images were captured with the animals in the supine position using a fluoroscopic imaging intensifier (DHF-105CX, Hitachi, Hitachi, Japan) under continuous 2.0–2.5% isoflurane inhalation. DHI was calculated and normalized to the pre-transplantation DHI, as previously described [49], with rat identities blinded to the investigators.

### 4.7. Disc Explantation and Processing

At 12 weeks, the animals were euthanized via 5% isoflurane overdose, tails were dissected, and the experimental discs were explanted following skin removal and separation from the contiguous vertebral bodies along the axial plane. Thus, functional spinal unit specimens were fixed in 10% (*v*/*v*) formalin for 4 days and decalcified by KyodoByori service (Kobe, Japan). Functional spine units were sectioned along the sagittal plane, photographed, and evaluated using the Thompson grading system [63] in a blinded manner.

### 4.8. Histological Assessment

Tissue specimens were processed in paraffin and sectioned at 4–5 µm slices, and subsequently stained with standard hematoxylin/eosin and safranin-O/fast-green staining using KyodoByori service (Kobe, Japan). Histopathological scoring was applied using the rat-specific ORS spine histological grading scheme [64], with assessments performed by blinded investigators. 

In the 10 samples with the lowest in the ORS spine histological grading (OLD *n* = 4, YOUNG *n* = 6) and healthy controls, human NP cells were traced through HLA-ABC staining by KyodoByori service (Kobe, Japan). In short, samples were deparaffinized and subjected to (i) 20 µg mL^−1^ proteinase/Tris-HCl (pH 7.6) for 10 min at 37 °C and (ii) 3.0 IU mL^−1^ hyaluronidase for 1 h at 37 °C. Endogenous peroxidase activity was tempered through 0.3% H_2_O_2_ incubation, followed by blocking using 3% bovine serum albumin and staining with purified monoclonal IgG1 anti-human HLA-ABC (BD Pharmingen, cat no 555551) at 1:50 dilution overnight. Sections were washed and subsequently stained using an anti-mouse IgG HRP-conjugated antibody for 1 h. Samples were then washed and stained with Simple Stain 3,3′-diaminobenzine solution, and counterstained with hematoxylin. Final histological and IHC stains were captured with a KEYENCE fluorescence microscope BZ-9000 (Keyence Ltd., Osaka, Japan) and digitally merged via imaging stitching. A negative control lacking the primary antibody was used to determine staining specificity.

### 4.9. Statistic Analysis, Randomization, and Data Presentation

Continuous data are presented as mean ± standard deviation and categorical data as frequency and/or percentage, unless otherwise specified. Data normality was confirmed through the Shapiro–Wilk test for all datasets, except for ordinal data (e.g., histological scores). In vitro comparisons involving YOUNG vs. OLD independent groups were analyzed with a two-tailed unpaired *t*-test. In vivo data were analyzed using a two-way ANOVA with Tukey’s post-test for multiple comparisons considering the different treatments and different time points as the two independent variables. Ordinal ranked data for macroscopic and histological scores were evaluated through a Kruskal–Wallis assessment corrected by Dunn’s test. Pearson correlation coefficients were calculated to test for the correlations between the variables age, proteoglycan positivity, COL2 positivity, COL1 positivity, spheroid CFU number, fibroblastic CFU number, total CFU number, viability rate, proliferation rate, CD24 positivity, GD2 positivity, and Tie2 positivity. The results are shown as a correlation matrix. This method was selected to investigate linear relationships among variables within our dataset derived from six different donors, highlighting the relation of specific cellular features. An alpha level of 0.05 was chosen for all relevant statistical tests. A *p*-value below 0.05 was considered statistically significant. All data were analyzed using GraphPad Prism v10 (GraphPad Software Inc., Boston, MA, USA). The “Rand” function in Microsoft Excel (version 16.78.3, Microsoft Corp) was employed to randomize treatment allocation to the different experimental discs. Blinded histological images and radiographs were prepared by an administrative staff member not involved in the assessment of study outcomes. Illustrations were made using Adobe Illustrator version 27.8.1 (Adobe Inc., San Jose, CA, USA).

## Data Availability

All data are presented in the manuscript. Additional data can be requested from the corresponding authors upon reasonable request.

## Abbreviations

CFA—Colony formation assay, CFU—Colony-forming unit, Co—Coccygeal level, COL1—Type I collagen, COL2, Type II collagen, CS10—CryoStor^®^-10, DHI—Disc height index, DMEM—Dulbecco’s Modified Eagle Medium, ECM—Extracellular matrix, FBS—Fetal bovine serum, FGF2—Fibroblast growth factor 2, GD2—Disialoganglioside, HA—Hyaluronate, HLA—Human leukocyte antigen, IHC—Immunohistochemistry, IVD—Intervertebral disc, LBP—Low back pain, LCS—Lumbar canal stenosis, LDH—Lumbar disc herniation, MEMα—Minimal essential medium α, MSC—Mesenchymal stromal cell, NP—Nucleus pulposus, NPPC—Nucleus pulposus progenitor cell, OLD—Cells obtained from older donors (>60 years old), ORS—Orthopedic Research Society, Tie2—Angiopoietin-1 receptor tyrosine kinase, YOUNG—Cells obtained from young donors (<25 years old), WTC—Whole tissue culture.
